# Diet Quality Trajectories From Infancy to Young Adulthood: The Special Turku Coronary Risk Factor Intervention Project (STRIP) Study

**DOI:** 10.1016/j.tjnut.2025.05.005

**Published:** 2025-05-12

**Authors:** Saija Tarro, Jussi Vahtera, Jaana Pentti, Harri Niinikoski, Olli Raitakari, Tapani Rönnemaa, Jorma Viikari, Katja Pahkala, Hanna Lagström

**Affiliations:** 1 Department of Public Health, University of Turku and Turku University Hospital, Turku, Finland; 2 Centre for Population Health Research, University of Turku and Turku University Hospital, Turku, Finland; 3 Clinicum, Faculty of Medicine, University of Helsinki, Helsinki, Finland; 4 Research Centre of Applied and Preventive Cardiovascular Medicine, University of Turku, Turku, Finland; 5 Department of Pediatrics and Adolescent Medicine, Turku University Hospital, University of Turku, Turku, Finland; 6 Department of Clinical Physiology and Nuclear Medicine, Turku University Hospital, Turku, Finland; 7 InFLAMES Research Flagship, University of Turku, Turku, Finland; 8 Department of Medicine, University of Turku, Turku, Finland; 9 Division of Medicine, Turku University Hospital, Turku, Finland; 10 Paavo Nurmi Centre and Unit for Health and Physical Activity, University of Turku, Turku, Finland; 11 Nutrition and Food Research Center, Faculty of Medicine, University of Turku, Finland

**Keywords:** diet quality, trajectory, neighborhood socioeconomic status, socioeconomic disadvantage, longitudinal birth cohort, children, adolescents, young adults

## Abstract

**Background:**

Stability in dietary habits has been observed during childhood and adolescence, but their stability from infancy to adulthood is less known.

**Objectives:**

Our aim was to identify latent diet quality trajectories from age 1 to 18 y and to examine their association with diet quality at age 26 y.

**Methods:**

The study included 620 participants from the Special Turku Coronary Risk Factor Intervention Project, initiated in infancy. Food and nutrient intake were assessed annually from age 1 to 18 y and again at age 26 y using food records. A food-based diet score (range: 0–33) was calculated to indicate diet quality. Group-based modeling was used to model trajectories of diet quality between the ages of 1 and 18 y. Logistic regression analysis examined associations of childhood sociodemographic characteristics with diet trajectories. Linear regression analyses investigated associations between the observed developmental diet quality trajectory groups and diet quality at age 26 y, adjusted for adulthood sociodemographic characteristics.

**Results:**

From age 1 to 18 y, 5 diet quality trajectory groups were identified: low (19% of participants), decreasing (25%), increasing (15%), intermediate (31%), and high (10%). Throughout the follow-up period, the diet score remained at 20–22 in the high diet quality trajectory group and at 11–13 in the low diet quality trajectory group. The diet quality trajectory groups predicted diet quality at age 26 y (*P* < 0.001). The adjusted mean difference in adulthood diet score between the low and high diet trajectory groups was 3.6 (95% CI: 1.5, 5.7). Notably, participants in the intervention group had higher scores than controls across all trajectories and throughout the entire follow-up period.

**Conclusions:**

The 5 distinct diet quality trajectory groups from infancy to adulthood highlight a clear difference between the highest and lowest diet quality groups. The findings suggest that dietary habits established in early childhood remain moderately stable into early adulthood.

## Introduction

Healthy dietary habits provide a basis for maintaining good health and preventing chronic diseases [1,2]. In early childhood, parents and the home environment play a key role in shaping dietary habits [3,4], and family resemblance in dietary habits is well documented [[5], [6], [7]]. In late adolescence and early adulthood, most individuals experience major transitions, such as leaving the parental home, leaving school to begin further education or paid employment, and starting a relationship. This transition period is characterized by increased independence, and it has been suggested to be an important age for the establishment of long-term health behaviors [8,9].

Previous studies examining the diet quality trajectories have observed stability of dietary scores over time from infancy to preschool age [10,11], from preschool age to middle childhood [12,13], and from middle childhood to early adolescence [14]. However, a gradual change in dietary patterns from early childhood to middle childhood have also been found [15]. Moreover, poor dietary habits established in adolescence tend to track into early adulthood [16]. However, most previous studies have had data for only 2 or 3 time points, and only a few studies have tracked dietary patterns from early childhood to adolescence [17] or adulthood [18,19]. To date, our understanding of whether dietary habits are maintained or subject to change between early childhood, adolescence, and adulthood is limited.

The experience of life transitions and subsequent changes in dietary habits may depend on the contexts in which the transition occurs, and different socioeconomic groups may experience these transitions differently. Socioeconomic status (SES) of the parents, the individuals themselves, and the residential neighborhoods are important factors that influence dietary habits throughout the life course [[20], [21], [22], [23], [24]]. It has been suggested that inequities in diet quality may increase over the life course, particularly in young adulthood [25].

The participants in the Special Turku Coronary Risk Factor Intervention Project (STRIP) [26] have been followed up from infancy to age 26 y. This allows a unique opportunity to investigate diet quality trajectories over time among the participants. The aim of this study was first to define latent diet quality trajectories from 1 to 18 y of age, i.e., up to age of majority, and to study how sex, parental SES, and neighborhood socioeconomic disadvantage in childhood are associated with diet quality trajectories. The second aim was to study the association between the observed diet quality trajectories and diet quality at age 26 y, taking into account individual-level and area-level socioeconomic characteristics in adulthood. The third objective was to investigate whether the infancy-onset dietary intervention involving a more heart-healthy diet had different effects on diet quality trajectories in the intervention and control groups.

## Methods

### Study design and participants

**FIGURE 1:**
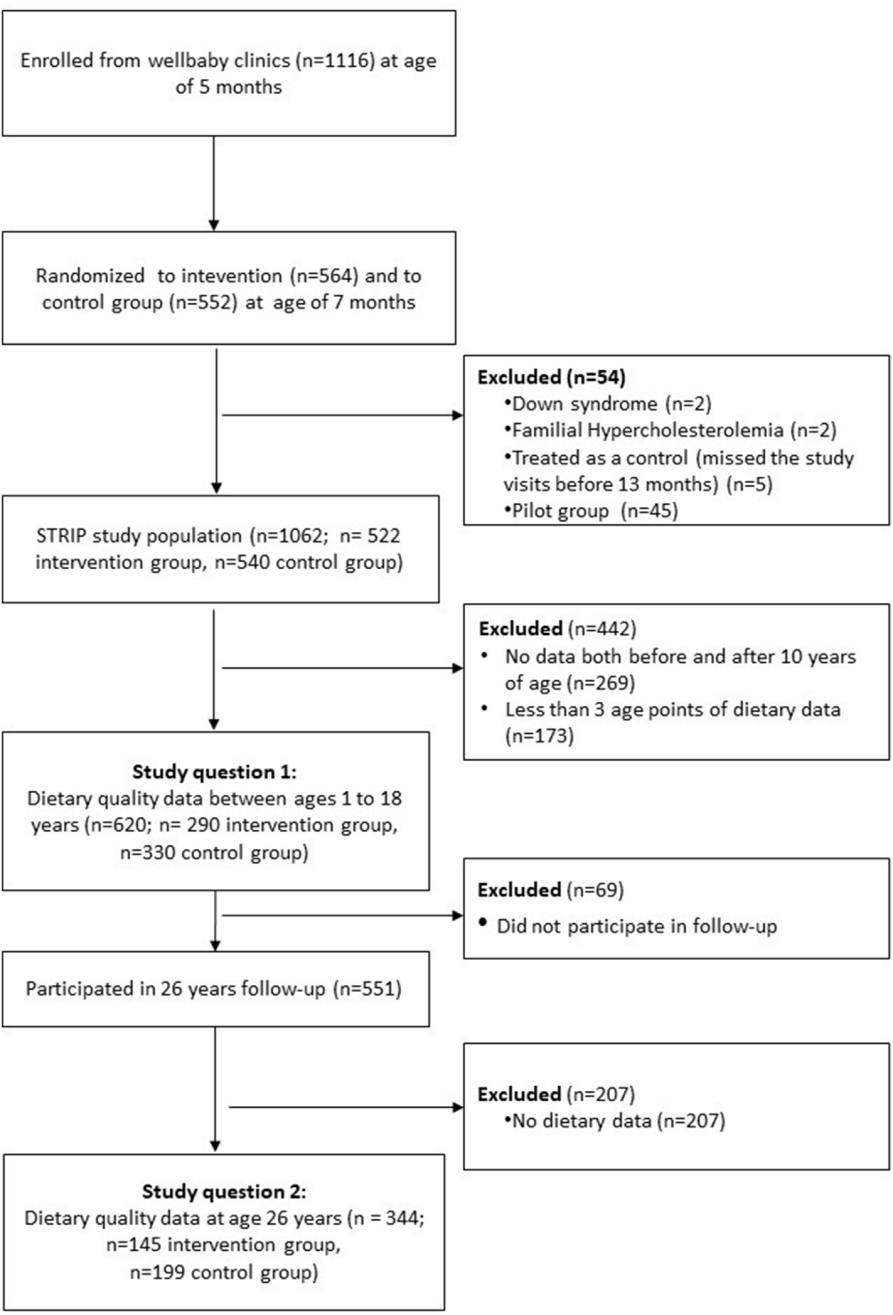
Flowchart summarizing the participants in this study.

This study is based on data from individuals participating in a prospective, randomized controlled cardiometabolic risk factor intervention project, the STRIP [26]. In STRIP, families of 5-mo-old infants (*N* = 1116 infants), born between July 1989 and December 1991 and recruited by nurses at well-baby clinics in Turku, Finland, were randomly allocated to either a dietary intervention (*n* = 564) or control (*n* = 552) groups. Both groups were followed up until the age of 20 y, and a postinterventional follow-up was done at the age of 26 y [27]. Individuals included in this study comprise those who provided dietary data at the age of 26 y and at least 4 measurements from 1 to 18 y of age (*n* = 620). We decided to investigate diet quality trajectories up to the age of majority, i.e., until adolescents are mostly still living at parental home. Of them, 47% belonged to the intervention group and 53% to the control group (Figure 1). Participants had an mean of 16.1 (SD: 2.4; range: 7–18) dietary measurements between ages 1 and 18 y.

The intervention group received individualized dietary counseling to reduce known cardiometabolic risk factors at 1-mo to 3-mo intervals from age 7 mo until the age of 2 y and twice per year thereafter until the age of 20 y [27]. The counseling was provided to parents until the child was aged 7 y, and thereafter, more information was gradually provided directly to the child. The control group children were seen twice per year until the age of 7 y and annually thereafter. The groups were followed up with similar measurements, including food diaries.

The STRIP study was approved by the Joint Commission on Ethics of Turku University and Turku University Central Hospital (Original Approval code: 32/2008, Approval date: 1 April 2008. 25-year follow-up study Approval code: TO5/028/2014, Approval date: 12 May 2014). Written informed consent was obtained from parents at study entry and from the participants at the ages of 15, 18, and 26 y.

### Measures

#### Dietary data

Dietary data were obtained close to each visit by using food records for 4 consecutive days, including 1–2 weekend days (3-d records before 2 y of age). The food record data were entered into the Micro-Nutrica food analysis software to calculate food and nutrient intakes (developed at the Research and Development Center of the Social Insurance Institution, Finland).

A food-based diet score was modified from the study by Nettleton et al. [28] for this study. Food intakes were classified into 7 favorable (fiber-rich grain products, fruits and berries, vegetables, fish, nuts and seeds, low-fat products, unsweetened dairy products, and vegetable oil–based fats) and 4 unfavorable food groups (red and processed meat, sugar-sweetened beverages, salty snacks, and desserts). Daily intakes of foods and drinks were calculated in grams per energy intake and then classified into quartiles: favorable foods were given ascending values (0, 1, 2, and 3) and unfavorable foods descending values (3, 2, 1, and 0). The values from each category were summed to create a favorable diet score and an unfavorable diet score. Higher values in both categories indicated better diet quality. In addition, the overall diet score (values from 0–33) was created by summing all categories together [29].

#### Neighborhood socioeconomic disadvantage

Data regarding neighborhood SES was derived from a grid database established and maintained by Statistics Finland [30]. The database contained spatiotemporal socioeconomic information from each residential area at a spatial resolution of 250 m × 250 m in between years 1990 and 2014. The neighborhood disadvantage score used is based on the proportion of adults with low education, the unemployment rate, and household income in each 250- × 250-m grid area [31]. For each of the 3 variables, we derived a standardized *z*-score based on the total Finnish population (mean: 0; SD: 1). A score for neighborhood disadvantage was then calculated by taking the mean value across the 3 *z*-scores. Higher scores on the continuous index denote greater disadvantage. For the statistical analyses, the neighborhood disadvantage score was classified into 2 categories based on national means as follows: ≤0 SD (low disadvantage/affluent) and >0 SD (high disadvantage).

High-quality residential mobility data, based on a complete history of the residential addresses with latitude and longitude coordinates, were obtained from the Population Register Center for each participant. Using open-source Geographical Information Systems (QGIS; http://www.qgis.org/en/site/), data on the residential neighborhood disadvantage for each time point were linked to the cohort participants’ home addresses by the latitude and longitude coordinates.

#### Sociodemographic characteristics

Sociodemographic characteristics in childhood were measured from parents’ responses to questionnaires at child age of 1, 5, and 9 y and by the level of cumulative neighborhood disadvantage until age 9 y. Parental education was classified into 2 categories, low and high, based on the highest education that one of the parents had completed for their professions. Those who had no professional training or a maximum of and intermediate level of vocational training were classified as “low.” Those who had studied at a university of applied sciences or higher, were classified as “high.” The high level included any academic degree (bachelor’s, master’s, licentiate, or doctoral degree). Parental occupation status was classified as manual and nonmanual occupation (including higher grade and intermediate occupations). Childhood cumulative socioeconomic disadvantage was calculated by taking the mean of the neighborhood disadvantage score from birth to age 9 y.

Sociodemographic characteristics in early adulthood were assessed with a self-administered questionnaire at the age of 26 y (postinterventional follow-up) and the level of neighborhood disadvantage between 19 and 26 y of age. Level of own education was classified into 2 categories (low/high education). Those participants who had no professional training or vocational studies were classified as “low.” Those who had a degree from university or university of applied sciences were classified as “high.” Work status was classified into 2 categories (no, i.e., students and those outside of workforce; or yes, i.e., employees or entrepreneurs) as was marital status (single/married or cohabiting). Adulthood cumulative socioeconomic disadvantage was calculated by taking the mean of the neighborhood disadvantage score between 19 and 26 y of age.

### Statistical analysis

The covariates were reported as means and SD or as absolute numbers and percentage, when appropriate. Group-based trajectory modeling (GBTM) was used to investigate the developmental trajectories (the evolution of an outcome over age) of diet score. Diet score trajectories were analyzed separately for the whole study population and for intervention and control groups. GBTM is a form of finite mixture modeling for analyzing longitudinal repeated-measure data [32]. In the model, participants were assigned to latent trajectory subgroups on the basis of their observed dietary habits during the study period of 18 y. The GBTM approach is flexible, allowing for partially missing data, and participants are assigned to a subgroup based on their highest posterior probability. Moreover, covariates can be also included in the model [33].

The GBTM analyses were conducted for all participants and for participants in the intervention and control groups using proc traj procedure in the SAS software (version 9.4; SAS Institute). The goodness-of-model fit was judged by fit-criteria assessment plot [34] in the Rstudio software (version 2023.09.0; RStudio). The cutoff for the smallest group was set at >5% of the entire cohort. The detailed description of the fit statistics and parameter estimates of the model are given in the Supplemental Figure 1. Diet score was treated as continuous variable.

Logistic regression analysis was used to examine the relationships of childhood sociodemographic characteristics (sex, parental education, parental occupation status, and neighborhood disadvantage in childhood) and intervention/control group with the diet trajectories. The results are reported as odds ratios (ORs) with the lowest diet quality group as the reference group.

Linear regression analyses were used to predict diet quality (continuous score) at age 26 y by the observed developmental diet quality trajectories. The models were adjusted with adulthood sociodemographic characteristics—sex, marital status, education level, work status, and neighborhood disadvantage—separately and in combination. To examine whether the associations varied between sociodemographic subgroups, we added the interaction term sociodemographic characteristic × diet quality trajectory to the model. The results are reported as mean differences between trajectories with 95% CI for all participants and for participants in the intervention and control groups.

## Results

The characteristics of the study population are shown in Table 1. During the first 9 y of life, the majority of children lived in affluent neighborhoods (64%), and their parents had a high education level (69%) and a nonmanual occupation (72%). No differences were found between the intervention and control participants in terms of sex, neighborhood socioeconomic disadvantage, parental occupation, and education.

**TABLE 1:** Descriptive characteristics of the study participants by intervention/control group.

Characteristic		All (*N* = 620)	Intervention (*n* = 290)	Control (*n* = 330)	*P*^1^
Sex	Female	49 (302)	48(139)	49 (163)	0.72
	Male	51 (318)	52 (151)	51 (167)	
Childhood (age 1–9 y)					
Parental occupation^2^	Manual	26 (164)	24 (69)	29 (95)	0.17
	Nonmanual	72 (445)	74 (215)	70 (230)	
	Missing	2 (11)	2 (6)	1 (5)	
Parental education level^2^	Low	29 (179)	26 (74)	32 (105)	0.12
	High	69 (429)	71 (207)	67 (222)	
	Missing	2 (12)	3 (9)	1 (3)	
Neighborhood disadvantage^3^	High (deprived)	36 (225)	34 (98)	38 (127)	0.23
	Low (affluent)	64 (395)	66 (192)	62 (203)	
Adulthood (age 26 y)		All (*n* = 344)	Intervention (*n* = 145)	Control (*n* = 199)	*P*^1^

Education level^4^	Low	29 (100)	29 (42)	14 (58)	0.91
	High	67 (232)	68 (99)	67 (133)	
	Missing	3 (12)	3 (4)	4 (8)	
Marital status	Single	44 (151)	42 (61)	45 (90)	0.51
	Married/cohabit	53 (182)	55 (80)	51 (102)	
	Missing	3 (11)	3 (4)	4 (7)	
Work status	No	44 (152)	47 (68)	42 (84)	0.45
	Yes	53 (182)	51 (74)	54 (108)	
	Missing	3 (10)	2 (3)	4 (7)	
Neighborhood disadvantage^5^	High (deprived)	38 (131)	41 (54)	59 (77)	0.78
	Low (affluent)	62 (213)	43 (91)	57 (122)	

The values are % (*n*).

1 *P* values from χ^2^ test for comparisons between intervention and control groups.

2 Family occupation and education from 1, 5, or 9 y of age. Education was classified into 2 categories: low and high education based on the highest education that 1 of the parents had completed for their professions. Those who had no professional training or a maximum of and intermediate level of vocational training were classified as “low.” Those who had studied at a university of applied sciences or higher were classified as “high.” Parental occupation status was classified as manual and nonmanual occupation (including higher grade and intermediate occupations).

3 Neighborhood socioeconomic disadvantage 0–9 y.

4 Level of own education at 26 y of age. Those participants who had no professional training or vocational studies were classified as low. Those who had a lower university degree (bachelor’s degree) or a degree from university of applied sciences were classified as “intermediate.” Those who had a higher university degree (master’s degree or higher) were classified as “high.”

5 Neighborhood socioeconomic disadvantage 19–26 y.

At the age of 26 y, most participants lived in affluent neighborhoods (62%), had a high education level (67%), were married or cohabiting (53%), and were employed (53%). No differences were found between intervention and control participants in terms of neighborhood socioeconomic disadvantage, education, marital status, and work status.

### Diet quality trajectories

**FIGURE 2:**
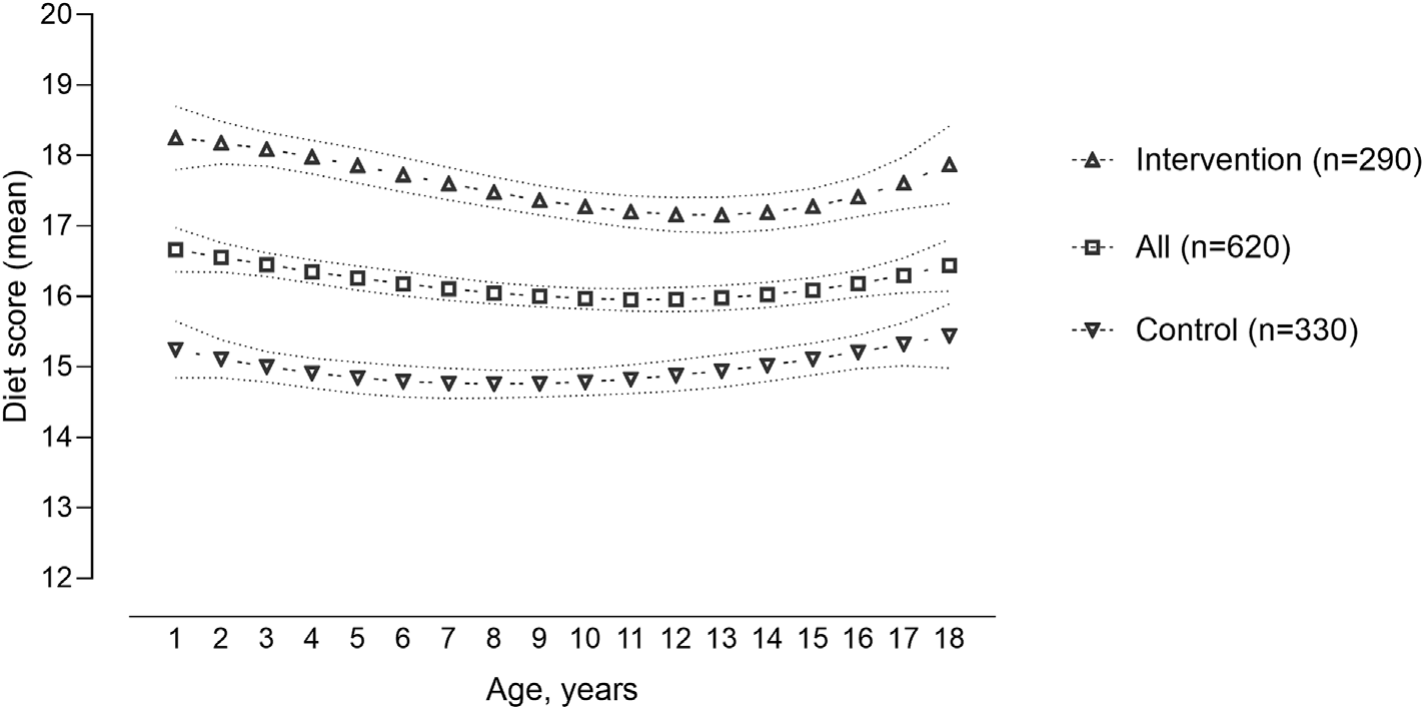
A single-trajectory model of diet quality score from 1 to 18 y of age for all participants and separately for the intervention and control participants.

The single-trajectory model (Figure 2) shows that there was a small dip in the diet score in adolescence, which levels off by age 18 y. Additionally, the mean diet score in the intervention group (18.1; SD: 4.8) was 3.1 points higher than that in the control group (15.0; SD: 5.2; *P* < 0.001).

The trajectory analyses suggested 5 types of diet quality trajectories as the optimal solution to characterize the whole study population over the 18-y period (Supplemental Figure 1). The smallest posterior probability was good (0.82) for the 5-group model. Compared with models with more groups, the model with 5 groups showed sufficiently good Bayesian information criterion and Akaike information criterion values (−28.352 and −28.419, respectively).

**FIGURE 3:**
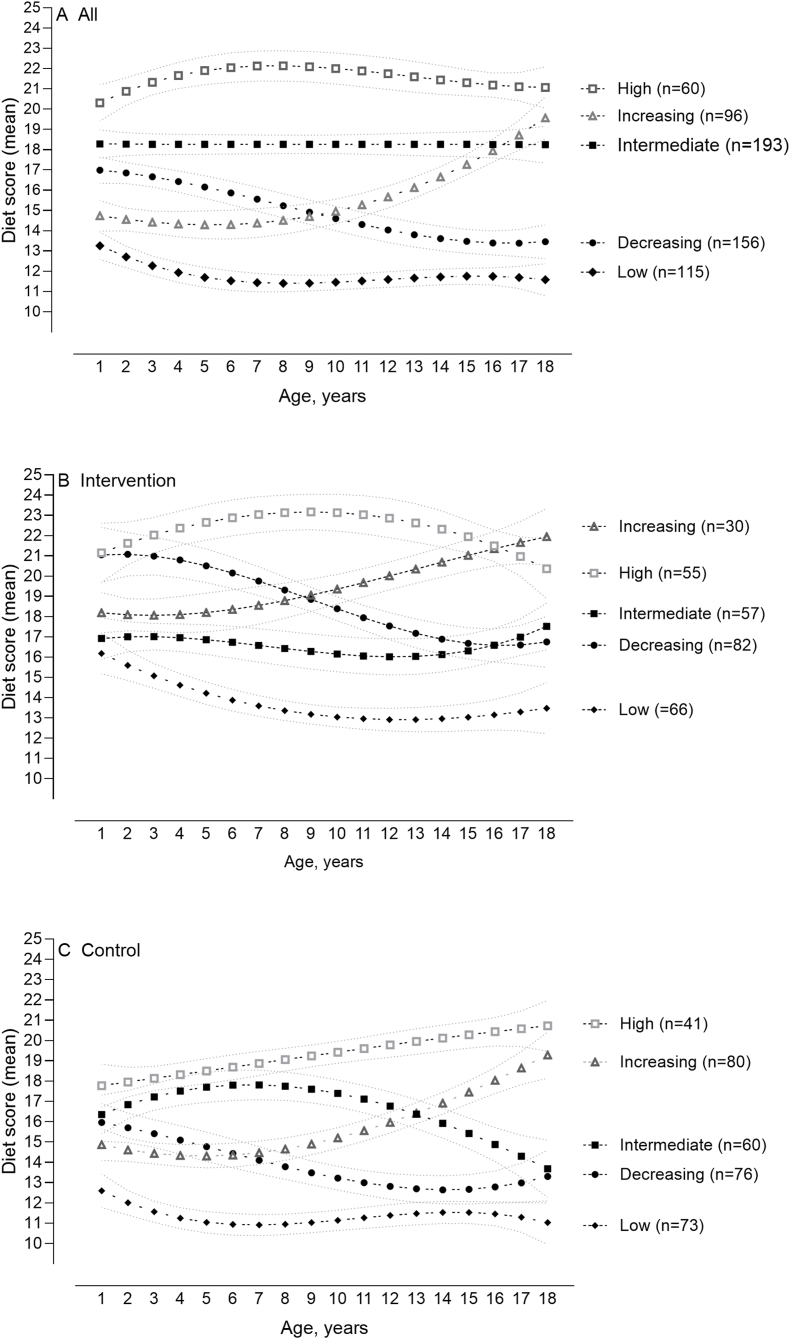
Group-based trajectory models identifying 5 distinct developmental trajectories of diet quality from 1 to 18 y of age of all (A), intervention (B), and control (C) participants.

As shown in Figure 3A, the 5-category trajectory included 3 groups that maintained a constant diet quality level throughout the follow-up period: high (10% of participants), intermediate (31%), and low (19%). In addition, increasing (15%) and decreasing (25%) trajectory groups were identified. Throughout the follow-up period, the diet score remained at the level of 20.3–22.1 in the high, at the level of l8.2–18.3 in the intermediate, and at the level of 11.4–13.3 in the low diet quality trajectory groups. The diet score rose from 14.3 to 19.6 in the increasing and fell from 17.0 to 13.4 in the decreasing trajectory groups. Figure 3 also shows the 5-category solution separately for intervention (Figure 3B) and control (Figure 3C) participants. Among the intervention participants, diet quality scores were consistently higher in each trajectory group than those in control participants in the corresponding trajectory group.

### Associations of diet quality groups with sociodemographic characteristics

Associations between childhood sociodemographic characteristics and diet quality trajectory groups are presented in Table 2. Compared with the low diet quality trajectory group (reference), there were more females in the other diet quality trajectory groups (OR: 1.7–5.0), and they were more likely to belong to the intervention group (OR: 3.8–23.8). Children in the increasing, intermediate, or high diet quality trajectory groups were more likely to have parents with high education (OR: 1.7–4.0) and nonmanual occupation status (OR: 3.1–3.7). Neighborhood socioeconomic disadvantage in childhood was not associated with diet quality trajectory groups.

**TABLE 2:** Associations between childhood sociodemographic characteristics and diet quality trajectories.

Characteristics	Trajectory group				
	Low, OR (ref)	Decreasing, OR (95% CI)	Intermediate, OR (95% CI)	Increasing, OR (95% CI)	High, OR (95% CI)
Girl vs boy	1	1.7 (1.0, 2.9)	3.2 (2.0, 5.3)	5.0 (2.8, 8.9)	2.8 (1.5, 5.4)
Intervention vs control participants	1	3.8 (2.1, 6.7)	8.4 (4.8, 14.7)	2.0 (1.0, 3.8)	23.8 (10.3, 54.6)
Neighborhood SES: low vs high^1^	1	0.9 (0.6, 1.5)	1.1 (0.7, 1.8)	0.9 (0.5, 1.6)	1.3 (0.7, 2.6)
Parental education: high vs low^2^	1	1.7 (1.0, 2.9)	2.5 (1.5, 4.1)	1.8 (1.0, 3.3)	4.0 (1.8, 9.0)
Parental occupation: nonmanual vs manual^2^	1	2.9 (1.7, 4.9)	3.7 (2.2, 6.2)	3.1 (1.7, 5.7)	3.5 (1.7, 7.3)

The values are ORs and their 95% CIs with the lowest diet quality group as the reference derived from logistic regression models.

OR, odds ratio; SES, socioeconomic status.

1 Neighborhood socioeconomic disadvantage 0–9 y.

2 Family occupation and education from 1, 5, or 9 y of age. Education was classified into 2 categories: low and high education based on the highest education that 1 of the parents had completed for their professions. Those who had no professional training or a maximum of and intermediate level of vocational training were classified as “low.” Those who had studied at a university of applied sciences or higher were classified as “high.” Parental occupation status was classified as manual and nonmanual occupation (including higher grade and intermediate occupations).

### Predicting diet quality at 26 y of age

Table 3 shows the mean level of the dietary score at the age of 26 y by the trajectory group for all participants, as well as separately for the intervention and control participants. The 18-y diet trajectory groups significantly predicted diet quality at age of 26 y (*P* < 0.001). The order of the end points of the trajectory groups at age 18 y persisted to age 26 y. Among all participants, adulthood diet score was 15.6 (SD: 4.7) for the low, 16.4 (SD: 5.3) for the decreasing, 19.3 (SD: 4.0) for the intermediate, 20.3 (SD: 5.3) for the increasing, and 20.8 (SD: 4.7) for the high diet quality trajectory group. Compared with the low diet trajectory group, the mean differences in diet scores at age 26 y were 3.6 points higher in the intermediate, 4.7 in the increasing, and 5.1 in the high trajectory groups (all *P* < 0.001). The association between trajectory groups and diet quality at 26 y remained significant after adjustments for sex, marital status, education level, work status, and adulthood neighborhood disadvantage. No differences in these associations by sex, marital status, or SES were observed (all *P*-interaction > 0.14). The results were essentially the same when studying the intervention and control participants separately (Table 3).

**TABLE 3:** Associations between developmental diet quality trajectory groups from 1 to 18 y of age and diet quality at age 26 y.

	Trajectories		Diet quality		Model 1^2^		Model 2^3^		Model 3^4^	
	Group	% (*n*)	Mean score^1^	95% CI	Mean difference	95% CI	Mean difference	95% CI	Mean difference	95% CI
All (*N* = 620)	Low	19 (115)	15.6	14.4, 16.9	Ref		Ref		Ref	
	Decreasing	25 (156)	16.4	15.5, 17.4	0.8	−0.8, 2.4	0.3	−1.2, 1.9	0.3	−1.2, 1.9
	Intermediate	31 (193)	19.3	18.4, 20.2	3.6	2.1, 5.1	2.2	0.7, 3.7	2.2	0.7, 3.7
	Increasing	15 (96)	20.3	19.1, 21.5	4.7	3.0, 6.4	3.2	1.6, 4.8	3.3	1.6, 4.9
	High	10 (60)	20.8	18.9, 22.6	5.1	2.9, 7.3	3.7	1.6, 5.8	3.6	1.5, 5.8
Intervention (*n* = 290)	Low	23 (66)	16.4	14.9, 17.9	Ref		Ref		Ref	
	Decreasing	28 (82)	19.1	17.8, 20.4	2.7	0.7, 4.7	2.4	0.5, 4.3	2.4	0.5, 4.3
	Intermediate	20 (57)	20.6	18.8, 22.3	4.1	1.8, 6.4	3.5	1.4, 5.8	3.6	1.4, 5.8
	Increasing	19 (55)	19.6	18.0, 21.3	3.2	1.0, 5.4	2.4	0.3, 4.6	2.4	0.3, 4.6
	High	10 (30)	20.7	18.1, 23.2	4.2	1.3, 7.2	3.5	0.7, 6.2	3.4	0.7, 6.2
Control (*n* = 330)	Low	22 (73)	15.4	13.8, 16.9	Ref		Ref		Ref	
	Decreasing	23 (76)	16.0	14.6, 17.4	0.6	−1.5, 2.7	0.2	−1.7, 2.2	0.2	−1.7, 2.2
	Intermediate	18 (60)	17.0	15.3, 18.7	1.2	−0.7, 3.9	−0.5	−2.7, 1.7	−0.6	−2.8, 1.7
	Increasing	24 (80)	20.1	18.8, 21.4	4.7	2.7, 6.7	2.9	0.9, 4.9	2.9	0.9, 4.9
	High	12 (41)	20.2	18.4, 22.0	4.9	2.5, 7.2	2.2	−0.2, 4.5	2.1	−0.3, 4.4

The values are mean differences and their 95% CIs with the lowest diet quality group as the reference derived from linear regression models.

1 Observed at age 26 y.

2 Model 1: base model.

3 Model 2: adjusted for sex, education level, marital status, and work status.

4 Model 3: adjusted for sex, education level, marital status, work status, and neighborhood disadvantage status.

## Discussion

In this study, we observed distinct developmental diet quality trajectory groups from infancy to age 18 y. We also found that childhood sociodemographic characteristics, such as sex and parental education, was associated with diet quality trajectories and that diet quality trajectory groups predicted diet quality at age 26 y.

By applying latent class modeling approach to annual dietary data from age 1 to 18 y, 5 distinct diet quality trajectory groups were found, with a marked difference between groups with the highest and lowest diet quality. Three of the identified diet quality trajectory groups remained moderately stable throughout the follow-up period and included more than half of the participants. The increasing trajectory group including 15% of the participants was almost at the same level as in the low trajectory group at the beginning of the follow-up, with a horizontal trend ≤9 y and upward trend thereafter. However, a quarter of the participants belonged to the decreasing trajectory group, characterized by a steady decline in diet quality from childhood to early adulthood. In the decreasing trajectory group, diet quality was almost at the same level as in the intermediate trajectory group at the beginning of the follow-up but declined steadily throughout the follow-up period, remaining below the level of the increasing trajectory group from age 9 y onward. This partially supports previous findings that a potential window of change is estimated to occur over 7–9 y, partly due to the influence of peers and other environmental factors [8,17].

The results of this study support the earlier findings from the STRIP study. We previously reported that the intervention participants had higher mean diet quality scores than the control group at 1–20 y of age [29]. Furthermore, there were no differences in mean nutrient intakes between the intervention and control families at baseline [35]. The possibility to study the effects of dietary intervention in the trajectories add a special feature to this study. The mean (single) trajectory was 2–3 dietary scores higher in the intervention group than that in the control group over the whole period, and this difference was observed in all 5 trajectory groups. Although the differences in the overall diet score were generally modest, the beneficial impact over 20 y suggest that dietary behavior can be influenced to promote health [29]. Most other dietary intervention trials have lasted only a few years [36,37], and the comparison of the results is challenging. In this study, we found that there was a slight dip in diet score in adolescence, which leveled off again by the age of 18 y. Similarly, a large United States cohort study showed that intakes of vegetables, whole fruit, and whole grains improved from adolescence to young adulthood [38].

When examining the association between childhood sociodemographic characteristics and diet quality trajectory groups, we found that parental education and occupation were associated with trajectory groups. This is consistent with earlier findings [10,39], suggesting that children whose mothers have the highest level of education are more likely to have a good diet quality than children with low educated parents. In these data, neighborhood SES was not associated with diet quality trajectory groups. In another Finnish study, children growing up in disadvantaged neighborhoods exhibited a trajectory of increasing BMI scores starting at 4 y of age, ending up with a higher risk of obesity by school age, than children living in more affluent neighborhoods [40]. The differences may be explained by the fact that childhood overweight is influenced by many factors other than diet quality, such as total caloric intake and amount of physical activity.

We also found that diet quality trajectory groups predicted diet quality in adulthood, at age 26 y. This is consistent with previous research findings, suggesting that eating habits develop during the early years and may continue into adulthood [18]. We observed small changes in diet score level between 18 and 26 y, but the order of the diet trajectory groups remained the same. The groups with lowest diet quality improved their diet quality, and the differences in diet quality narrowed compared with the end point of the trajectories. Previous studies have highlighted the importance of educational attainment for diet quality in adulthood [21,41], whereas less support has been found for the effect of beginning to cohabit on diet quality [42,43]. In our study, the association between trajectory groups and diet quality at 26 y attenuated partially but remained marked after adjustments for sex, marital status, education level, work status, and adulthood neighborhood disadvantage.

Assessing the diet quality of same participants from toddlerhood to adulthood and using the same dietary assessment method consistently throughout the follow-up period is the major strength of our study. It is rare to have records of eating habits over this developmental period. Second, taking into account the heterogeneity in diet quality patterns during the follow-up period using GBTM is another strength of the study. Limitations of this study include self-reported food records, which are prone to underreporting of unhealthy foods and overreporting of healthy foods. Another limitation is possible selection bias due to selective drop out during the follow-up. However, the characteristics of those participating in the study and those lost to follow-up have been compared, and no differences have been found regarding dietary components and SES [26,27]. Further, we had information on the home addresses of the participants only, thus we do not know when the adolescents moved away from their parents.

In conclusion, 5 distinct diet trajectory groups were identified from infancy to adulthood, with a clear difference between the groups with the highest and lowest diet quality. In addition to the 3 stable diet trajectory groups, a large proportion of participants were in the group with declining diet quality. Our findings support the notion that dietary habits established in toddlerhood are moderately sustained into adulthood. Furthermore, dietary intervention initiated in early childhood was evident across all trajectories and persisted into adulthood. To promote healthy dietary patterns, child diet quality should be regularly assessed through questionnaires administered during routine follow-ups in well-baby clinics and school health services, with particular attention given to children whose diet quality is declining.

## Author contributions

The authors’ responsibilities were as follows – HN, OR, TR, JVi, KP, HL: designed the research; HN, KP, HL: conducted the research; ST, JP: analyzed the data; ST, JVa, JP, HN, OR, TR, JVi, KP, HL: wrote the paper; HL: had primary responsibility for final content; and all authors: have read and approved the final manuscript.

## Data availability

Owing to legal restrictions, the data from STRIP study cannot be stored in public repositories or otherwise made publicly available. The rights to the data belong to the STRIP research group. Data access may be permitted on a case-by-case basis upon request. Data sharing outside the STRIP group requires a data sharing agreement. Investigators can submit an expression of interest to the STRIP Steering Committee.

## Funding

This research was funded by the Research Council of Finland (grants 206374, 294834, 251360, 275595, 307996, 322112, 295741, and 321409), the Juho Vainio Foundation (grant 202300246), the Finnish Foundation for Cardiovascular Research, the Finnish Ministry of Education and Culture, the Finnish Cultural Foundation, the Sigrid Jusélius Foundation, Special Governmental grants for Health Sciences Research (Turku University Hospital), the Yrjö Jahnsson Foundation, the Finnish Medical Foundation, and the Turku University Foundation. The funding bodies played no role in the study design, in the collection, analysis, and interpretation of data; in the writing of the report; and in the any restrictions regarding the submission of the report for publication.

## Conflict of interest

The authors report no conflicts of interests.
