# Supplementary material for: Diet Quality Trajectories From Infancy to Young Adulthood: The Special Turku Coronary Risk Factor Intervention Project (STRIP) Study
Source: J Nutr. 2025 May 12;155(6):1923–32. doi: 10.1016/j.tjnut.2025.05.005 (PMC12264548; doi:10.1016/j.tjnut.2025.05.005)
Supplement: multimedia component 1 [file mmc1.docx]

**Diet quality trajectories from infancy to young adulthood: The STRIP Study.**

# Saija Tarro

# **Supplementary Figure 1.** Description of applied Group Based Trajectory Model (GBTM) and Fit-Criteria Assessment Plots (FCAP).

Group Based Trajectory Model (GBTM) (1,2) was run with one to ten diet quality group solutions to acquire an idea of the latent heterogeneity in the diet quality. Model selection was assisted by the following fit statistics: Akaike Information Criterion (AIC), Bayesian Information Criterion (BIC), likelihood (L), average posterior probability of assignment (APPA), odds of correct classification (OCC), mismatch between estimated and assigned probabilities and standard deviation of group membership probabilities using The Fit-Criteria Assessment Plot (FCAP) (3). FCAP shows how indices change by increasing the number of latent trajectories and the visual display allows for different criteria to be assessed side by side to reach the best decision (see fit-criteria assessment plots). Theoretical relevance was judged by visual inspection of the physical activity pattern groups and using the criterion that the smallest group should include at least 5% of the total study population. After choosing the preferred model, each participant is classified to one of the diet quality groups based on his/her maximum posterior probability of assignment to the extracted bivariate classes (see output of the GBTM model).

Fit-criteria assessment plots for trajectory modeling

Akaike’s information criterion, Bayesian information criterion and Likelihood

Posterior probability, mismatch and SD

Odds of correct classification

Percentage of individuals belonging to the smallest group

**Maximum Likelihood Estimates of the trajectory groups (SAS output)**

Maximum Likelihood Estimates

Model: Censored Normal (cnorm)

Standard T for H0:

Group Parameter Estimate Error Parameter=0 Prob > |T|

1 Intercept 13.93305 0.48579 28.681 0.0000

Linear -0.74465 0.21665 -3.437 0.0006

Quadratic 0.06908 0.02690 2.568 0.0103

Cubic -0.00194 0.00095 -2.047 0.0407

2 Intercept 17.06257 0.45990 37.100 0.0000

Linear -0.04336 0.20181 -0.215 0.8299

Quadratic -0.03473 0.02421 -1.435 0.1515

Cubic 0.00145 0.00085 1.704 0.0884

3 Intercept 14.96224 0.58609 25.529 0.0000

Linear -0.24913 0.28933 -0.861 0.3892

Quadratic 0.02078 0.03399 0.611 0.5410

Cubic 0.00040 0.00115 0.350 0.7261

4 Intercept 18.28762 0.46234 39.554 0.0000

Linear -0.01003 0.17546 -0.057 0.9544

Quadratic 0.00112 0.02163 0.052 0.9586

Cubic -0.00004 0.00076 -0.051 0.9595

5 Intercept 19.60946 0.64150 30.568 0.0000

Linear 0.77109 0.28448 2.710 0.0067

Quadratic -0.07143 0.03519 -2.030 0.0424

Cubic 0.00184 0.00125 1.466 0.1428

Sigma 3.84359 0.02788 137.876 0.0000

Group membership

1 (%) 17.97510 2.15416 8.344 0.0000

2 (%) 25.09606 2.69773 9.303 0.0000

3 (%) 16.63530 2.77716 5.990 0.0000

4 (%) 30.51278 2.80754 10.868 0.0000

5 (%) 9.78076 1.86011 5.258 0.0000

BIC=-28439.31 (N=9974) BIC=-28404.58 (N=620) AIC=-28349.21 L=-28324.21

**References**

1. Nagin D, Odgers C. Group-Based Trajectory Modeling in Clinical Research. Annual review of clinical psychology. Palo Alto, Calif. : Annual Reviews,; 2010. 109–138 p. (Annual Review of Clinical Psychology; vol. 6).
2. Nagin DS, Jones BL, Passos VL, Tremblay RE. Group-based multi-trajectory modeling. Stat Methods Med Res. 2018 Jul;27(7):2015–23.
3. Klijn SL, Weijenberg MP, Lemmens P, Van Den Brandt PA, Lima Passos V. Introducing the fit-criteria assessment plot-A visualisation tool to assist class enumeration in group-based trajectory modelling. Stat Methods Med Res. 2017 Oct;26(5):2424–36.
